# Resilient phenotypes among bereaved youth: a comparison of trajectory, relative, and cross-domain approaches

**DOI:** 10.1186/s13034-023-00568-0

**Published:** 2023-02-08

**Authors:** Ana Lucia Espinosa Dice, Xian Ye, Stephanie Gyuri Kim, Katie A. McLaughlin, Ananda B. Amstadter, Henning Tiemeier, Christy A. Denckla

**Affiliations:** 1 Department of Epidemiology, Harvard T.H. Chan School of Public Health, Boston, MA USA; 2 Department of Biostatistics, Harvard T.H. Chan School of Public Health, Boston, MA USA; 3 Human Development and Family Studies, University of Illinois Urbana-Champaign, Champaign, IL USA; 4 Department of Psychology, Harvard University, Cambridge, MA USA; 5 Virginia Institute for Psychiatric and Behavioral Genetics, Virginia Commonwealth University, Richmond, VA USA; 6 Department of Social and Behavioral Sciences, Harvard T.H. Chan School of Public Health, Boston, MA USA

**Keywords:** ALSPAC, Resilience, Bereavement, Latent growth mixture models, Childhood and adolescence

## Abstract

**Background:**

Bereavement is a common traumatic event associated with adverse health outcomes across the life course. Despite these risks, not all bereaved individuals experience these negative effects. Limited scientific consensus exists on how to define resilience in individuals who have experienced the death of a loved one.

**Methods:**

Using a sample of N = 3766 youth from the Avon Longitudinal Study of Parents and Children birth cohort, we identified bereavement of a family member between ages 7 and 8.5. We derived and compared three different approaches to assess resilience among bereaved youth. *Trajectory-based psychological resilience* identified sub-groups with similar psychological symptom profiles between ages 6 and 16 using latent growth mixture models. *Relative psychological resilience* at age 16 leveraged standardized residuals from a model regressing psychological symptoms on bereavement to determine better-than-expected psychological functioning relative to bereavement status. *Relative cross-domain resilience* around age 16 was a sum score of the residuals approach applied to eight unique domains of health. Predictive validity of each approach was assessed using depressive symptoms at age 17.5

**Results:**

Overall, N = 877 (23%) youth were bereaved of a family member between ages 7 and 8.5. Using latent growth mixture models, a three-class solution described 84% of bereaved youth with low and stable psychological symptoms over time, 8% with worsening symptoms, and 8% with improving yet elevated symptoms. Each relative resilience score was largely concordant with the trajectory-based approach in identifying individuals as resilient or not, though relative psychological resilience demonstrated a stronger degree of concordance than the cross-domain score. Relative psychological and cross-domain resilience exhibited moderate to low correlation, depending on the domains included (r = 0.14–0.43). For each approach, resilience significantly predicted lower depressive symptoms at age 17.5, highlighting predictive validity of these measures.

**Conclusions:**

Psychological symptom trajectories among bereaved youth aligned with those previously identified among bereaved adults. The residual-based approach to defining resilience exhibited limited utility in the context of bereavement. When identifying risk and resilience after bereavement, researchers and clinicians must address the interplay across psychosocial and physical health domains, as bereaved youth considered resilient from a mental health perspective may benefit from intervention in other domains.

**Supplementary Information:**

The online version contains supplementary material available at 10.1186/s13034-023-00568-0.

## Background

The death of a loved one is among the most common and impactful traumatic events reported globally [1, 2]. In line with extensive literature documenting the widespread impacts of adverse childhood experiences [3, 4], bereavement among children and adolescents associates with adverse psychosocial outcomes across the life course, including disruptions to social and familial support structures [5–7] and increased risk of substance use [8], depressive symptoms and behaviors [9–12], and sleep and school problems [12]. Bereavement during childhood is also associated with adverse physical health outcomes, including cardiometabolic concerns [13] and cortisol abnormalities [14]. The high risks for these negative outcomes are not constrained to the loss of a parent or primary caregiver but extend to the loss of first- and second-degree relatives and close friends as well [8, 9]. Despite the potential risks following bereavement, not all youth who face the loss of a loved one experience these adverse effects. Evidence suggests that most individuals who experience bereavement integrate grief without lasting adverse health outcomes [15, 16]. Resilience, or the ability to adapt or maintain healthy levels of functioning in the face of trauma exposure [16–18], is especially salient in the context of bereavement because of the high prevalence of this exposure and the multi-dimensional adverse impacts associated. Understanding resilience and risk is critical to better informing prevention and intervention efforts among bereaved youth.

A significant barrier to progress is that approaches to identifying and quantifying resilience proliferate with little scientific consensus on the relative advantages and disadvantages of each, especially among bereaved individuals [19–21]. Furthermore, understanding resilience in the context of bereavement presents a unique set of considerations that do not necessarily intersect with other traumatic exposures. First, the loss of a loved one can have direct impacts on structural factors (e.g., loss of familial income, changes in education setting induced by relocation) and the social ecosystem on which children depend (e.g., reduced social network). Second, post-bereavement pathogenesis may be distinguishable from other post-trauma conditions as evidenced by extant literature demonstrating unique syndromic profiles associated with grief symptoms specifically [22, 23]. Given the evidence that bereavement during childhood is likely to affect multiple health pathways, including biological regulatory systems, multidimensional evaluations of the impact of this common exposure are critical [24]. Finally, resilience is often defined by the absence of psychopathology, assuming homogeneity in adjustment to trauma and overlooking a more comprehensive state of well-being that includes both positive psychosocial functioning and physical health [25, 26]. Approaches commonly used to address these limitations employ methods such as latent growth mixture models (LGMMs), which suggest relative consistency in psychological outcome trajectories, with most individuals sustaining low symptoms over time following a common stressor [16, 19, 27]. Other approaches assume that resilience is continuously distributed and model resilience in terms of deviation from what is expected relative to trauma exposure status [28, 29]. However, congruence across approaches is not commonly investigated, resulting in limited comparability across studies and settings [21, 30, 31]. Additionally, given the narrow focus on psychological functioning and internalizing disorders in the extant literature, individuals who may be asymptomatic in some domains of well-being, but struggling to adjust to loss in other unmeasured domains, are not typically identified [32, 33].

In response, the present analysis develops and compares three resilience constructs on the same data to determine who is resilient under multiple definitions vs. one, with each construct informed by the strengths and limitations of existing approaches. We opt to compare different constructs given the lack of research about resilience among bereaved youth and the need for additional evaluation of different resilience constructs and related issues of misclassification. To address this, we vary (1) the domains of functioning or well-being through which we quantify resilience, (2) the timing of our outcome assessments, and (3) the assumptions underlying the distribution of resilience. Specifically, we construct trajectory-based psychological resilience, relative psychological resilience, and relative cross-domain resilience. The trajectory-based approach uses LGMMs to identify classes of bereaved youth with distinct longitudinal psychological symptom profiles. Relative psychological resilience draws on standardized residuals from a linear model regressing psychological symptoms on bereavement to determine who is doing better than expected given their bereavement status; relative cross-domain resilience is based on a sum score of the residuals approach applied to eight unique domains of health. We examine concordance and correlations between constructs as well as the predictive validity of each one. The aims of these analyses are to evaluate the performance of different resilience constructs using the same data among a bereaved youth sample, and to characterize how multiple domains of functioning and different assumed distributions of resilience result in potential misclassification of post-bereavement well-being.

## Methods

### Study population

The sample consisted of participants enrolled in the Avon Longitudinal Study of Parents and Children (ALSPAC), a birth cohort epidemiological study of parents and their children. All pregnant women resident in Avon, UK with expected delivery dates between April 1, 1991 and December 31, 1992 were invited to participate [34–36]. Of the 15,447 pregnancies enrolled, there were 15,658 foetuses, 14,901 of which were alive at one year of age. Detailed health and socio-demographic data were collected via self-, maternal-, and paternal-report questionnaires as well as in-person assessment clinics. Please note that the study website contains details of all the data that is available through a fully searchable data dictionary and variable search tool: http://www.bristol.ac.uk/alspac/researchers/our-data/.

At 8 years 7 months, 8,304 mothers of youth filled out a questionnaire that inquired about, among other information, the loss of a youth’s family member since the age of 7. Of those respondents, 8,195 mothers (99%) answered questions on the youth’s loss and thus were included in this analysis. See Additional file 1 for a flow chart of participant retention across analytic samples. Selection bias could occur at this stage of bereavement ascertainment if mothers of those who were bereaved were less likely to respond to the questionnaire at 8 years 7 months than mothers of those who were not bereaved. However, given our focus on child outcomes rather than parent outcomes and the fact that participation rate by mothers remained high until mid-late adolescence [37], we did not use inverse probability weighting in our analyses.

Among 8195 youth with bereavement status ascertained, 4191 participated in an ALSPAC clinic at age 17 (51% participation rate in this clinic did not differ by bereavement status). We further restricted analyses to these youth, as many of our outcomes were measured at this clinic. Finally, we restricted to 3766 youth with at least three Strengths and Difficulties Questionnaire and three Moods and Feelings Questionnaire measures across five timepoints. The latter step was used to ensure sufficient observed outcome data for sound imputation of outcomes and covariates (more details on imputation under Statistical Analysis); given that the proportions of bereaved and non-bereaved individuals who met this last criterion were similar (90% non-bereaved vs. 88% bereaved), we did not use inverse probability weighting in our analyses.

### Measures

#### Bereavement

At 8 years and 7 months, mothers reported on the death of a youth’s family member: “Since his 7th birthday… somebody in the family died.” Response options included: ‘Yes and he was very upset’; ‘Yes and he was quite upset’; ‘Yes and he was a bit upset’; ‘Yes but he wasn’t upset’; and ‘No did not happen.’ Relationship to person who died was not asked. To define bereavement for these analyses, responses to this question were recoded into binary loss versus no loss categories, avoiding reliance on qualitative judgments from someone other than the bereaved child to quantify the impact of this exposure [38].

#### Mental health

Symptoms of psychopathology were assessed using the Strengths and Difficulties Questionnaire (SDQ) [39]. The SDQ is a 25-item behavioral screening tool composed of four sub-scales capturing hyperactivity, emotional symptoms, conduct problems, and peer problems. It was administered to the mother/caregiver and completed on behalf of the study youth at the ages of 6, 9, 11, 13, and 16. Responses were recorded on a 3-point scale ranging from ‘not true’ to ‘certainly true.’ Item responses were reverse coded, and total scores were derived by summing item responses. Prorated scores–scores weighted according to the non-missing items for comparability to those based on fully observed data–were used if no more than 8 items were missing. If more than 8 items were missing, the score was set to missing. Reliability of the SDQ total difficulties score was high across administration waves at ages 6, 9, 11, and 13 (Cronbach’s alpha by time point: 0.76, 0.79, 0.79, 0.73). Reliability was not calculated for the SDQ at age 16 because item-level data were not available.

Symptoms of depression in late adolescence were assessed using the Short Moods and Feelings Questionnaire (MFQ) [40]. At 17.5 years, study adolescents were administered the MFQ through an online survey. Items responses in the ALSPAC questionnaires were on a three-point scale ranging from ‘true’ to ‘not true.’ Items were reverse coded such that higher scores indicated greater depression, and a total depression score ranging from 0 to 26 was derived by summing all items. As per the ALSPAC team’s derivation, scores were not prorated, instead requiring responses to all 13 questions. Cronbach’s alpha was 0.90, demonstrating excellent internal consistency in the current sample.

#### Alcohol use

Alcohol use was assessed using the Alcohol Use Disorders Identification Test (AUDIT) [41]. At 17, youth were administered the AUDIT, a 10-item questionnaire about alcohol related disorders. Questions assess frequency and quantity of alcohol consumption, dependence behaviors, and harm resulting from alcohol use. Total scores range from 0 to 40, with higher scores indicating unhealthy alcohol use. Reliability was not calculated for the AUDIT because item-level data were not available.

#### Functional status

School attendance/absenteeism was assessed as the frequency of school attendance. School absenteeism has been shown to correlate strongly with academic performance and is often studied as an outcome in and of itself [42, 43]. At 16, youth were asked to describe the percentage that they attended school, with response options including: none, 10%, 20%, 40%, 60%, 80%, 100%, or not registered. School attendance was transformed as a continuous measure, grouping ‘not registered’ and ‘none’ responses at 0%.

Social functioning was assessed as the number of self-reported close friends. At 17, youth were asked to report their number of close friends, with response options including: 0, 1, 2–4, 5–9, 10–13, 15–19, or 20 + . Number of friends was transformed as a continuous measure. For responses 0, 1, and 20 + , an individual was assigned values 0, 1, and 20, respectively. For response 2–4, an individual was randomly assigned a number between 2 and 4, with equal probability of each number being selected. This within-range random assignment was repeated for the remaining response options as well (5–9, 10–13, and 15–19).

Sleep duration was assessed using average amount of sleep on a school night. At 15.5 years, youth were asked about the length of time they sleep on a normal school night. Reports in hours and minutes were converted to hours with decimal values accepted.

#### Physical health

Cardiometabolic health was defined by both systolic blood pressure and body mass index (BMI). At 17, resting blood pressure was measured using a DINAMAP 9301 machine and was taken twice on each arm. In these analyses, we used the average of two measures for right arm systolic blood pressure, though if only one measure was taken, that was used. Body mass index (BMI) was derived from height and weight measurements as kg/m^2^. Height and weight were measured using a Harpenden stadiometer (to the last complete mm) and the Tanita Body Fat Analyzer (Model TBF 401A; to the nearest 50 g), respectively.

Finally, body inflammation was assessed by C-reactive protein (CRP) levels. At 17, CRP was measured in mg/l from a blood assay.

#### Resilience constructs

**Fig. 1:**
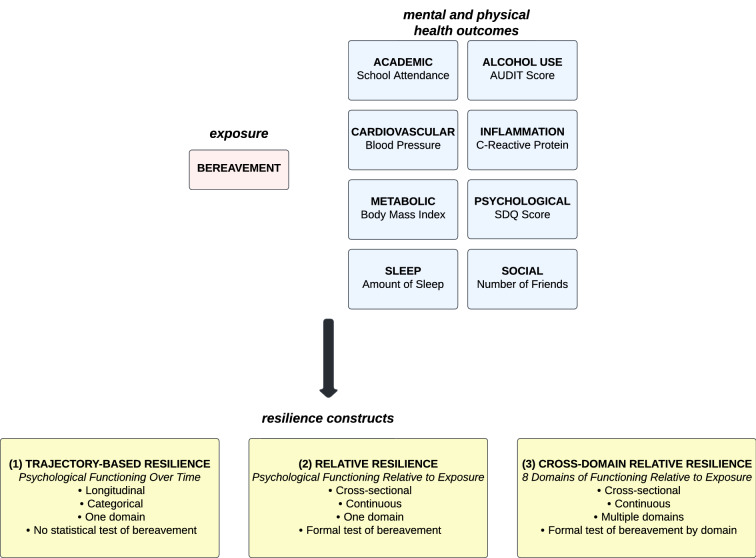
Schematic of three resilience constructs

We derived three different methods to define resilience: a trajectory-based approach, a single-domain relative resilience approach, and a cross-domain relative resilience approach. See Fig. 1 for a schematic of these constructs.

For the trajectory-based approach, we conducted latent growth mixture modeling (LGMM) to identify heterogeneous sub-populations with distinct psychological response patterns to bereavement. This approach has been widely utilized among bereaved adults, with results typically identifying two to five core response trajectories, including chronically elevated symptoms, acute symptoms followed by recovery, and sustained low symptoms [19, 20, 44]. In the present investigation, we modeled symptoms of psychopathology across time using the SDQ total difficulties scores from pre-loss (age 6) to post-loss (ages 9, 11, 13, and 16).

To calculate relative psychological resilience, we followed methods used previously among trauma-exposed adults [21, 28, 29] as well as in the ALSPAC sample specifically with regards to count of adverse childhood experiences [45]. We regressed SDQ total difficulties score at age 16 on bereavement and utilized the normed standardized residuals from that regression as an indicator of relative resilience. With this approach, a positive residual will reflect a healthier outcome than would be expected given bereavement exposure. A negative residual, on the other hand, would reflect a poorer outcome relative to this exposure. An advantage to this approach is that the resulting continuous score is specific to confederates in a specific sample population. We selected SDQ at age 16 (versus earlier timepoints) to harmonize with the cross-domain measures (see below) assessed around age 16. We selected SDQ over MFQ to optimize comparability with trajectory-based resilience, exploring two different ways to define resilience within one domain of health.

The third construct, what we call a relative cross-domain resilience score, defines resilience continuously and additively across eight domains of health assessed around age 16: psychological functioning, social functioning, school attendance, alcohol use, sleep duration, cardiometabolic health (BMI and blood pressure), and body inflammation. After obtaining normed standardized residuals from regressing each domain separately on bereavement, we summed these into a single cross-domain score, such that a higher score suggests better cross-domain resilience. Specifically, lower SDQ score, AUDIT score, systolic blood pressure, CRP, and BMI, as well as higher number of friends, school attendance, and sleep, reflected a positive health outcome. We considered more sleep to be positive given the higher amount of sleep deemed appropriate for children and adolescents (8–11 h) versus for adults (7–9 h) and the extremely low prevalence of sleep over 11 h reported in our analytic sample [46]. Additionally, we considered lower BMI to be positive given the relationship between BMI and cardiometabolic health that was of interest and the extremely low prevalence of underweight in our analytic sample [47].

#### Covariates

Sociodemographic covariates were measured during pregnancy, at birth, or before bereavement exposure. Child sex (male or female) was determined from the birth notification. Mother’s prenatal financial difficulties score (range 0–15) was assessed at 32 weeks’ gestation based on self-reported difficulties in affording the following items: food, clothing, heating, rent/mortgage, and baby items. Each item was ranked on a scale of 0 (not difficult to afford) to 3 (very difficult to afford). Mother’s highest educational attainment was self-reported at 32 weeks’ gestation. Father’s highest educational attainment was reported by the mother at this same questionnaire. Maternal smoking during pregnancy was considered present if she reported smoking one or more cigarettes in response to a query on current smoking status in a questionnaire sent between 24- and 41-weeks’ gestation. Quintiles of the Townsend deprivation score, a population measure of material deprivation, were derived based on a mother’s reported address during pregnancy. Finally, youth’s prior life events exposure between the ages of 5 and 7 was measured via maternal response to questions about 18 potentially upsetting events in the youth’s life (e.g., child admitted to hospital, child changed caretaker), assessing both event occurrence and child reaction on a 5-point scale from ‘did not happen’ to ‘yes, very upset.’ Items were coded such that higher scores indicated greater impact on child, and the total life events score ranging from 0 to 72 was derived by summing all items. This score was prorated, allowing at most half of components to be missing.

### Statistical analysis

To identify latent trajectories of psychopathology symptoms, captured by the SDQ total difficulties score between ages 6 and 16, using LGMM, we specified models following Jung and Wickrama [48]. First, we fit a single class growth model without covariates to ensure convergence. Second, we progressively fit models with two to six classes, assessing model fit according to a variety of indices: Akaike information criterion (AIC); Bayesian information criterion (BIC); sample-size adjusted Bayesian information criterion (SSBIC); and entropy, which identifies how clearly the model divides the classes. Two likelihood ratio tests were also used to adjudicate model fit: Lo-Mendell-Rubin likelihood test (LMR-LRT) [49] and Bootstrap Likelihood Ratio Test (BLRT). Third, covariates were added as predictors of latent class membership in a multinomial logistic regression. Step two was then repeated using conditional models since unconditional models could result in distorted results if covariates have strong effects on intercepts and class membership. Covariates, all numerically entered, include child’s sex (female vs. male), child’s life events score between 5 and 7, maternal financial difficulties at 32 weeks of gestation, paternal and maternal educational attainment, maternal smoking status around birth (yes vs. no), and Townsend deprivation index quintiles. Final model specification was determined by fit indices, interpretability of class assignment, and model parsimony. To facilitate model stability and proper class assignment that could be generalized to different datasets from targeted populations, we fixed the variance of the slope to zero as recommended in prior literature [48, 50]. Following model development and class assignment, each class was subjectively characterized based on mean class trajectories and categorized as resilient or non-resilient accordingly. We drew on the prototypical trajectories of adjustment following a potentially traumatic event outlined by Bonanno, whereby low symptoms, or positive adaptation (allowing for temporary disruptions to these patterns following trauma), are considered resilient [16, 27].

To define relative psychological resilience, we regressed SDQ total difficulties score at age 16 (square root transformed due to high skew) on bereavement, obtaining normed standardized residuals from that regression for each bereaved individual. As mentioned, we negated all residual values for better interpretability such that a positive residual would indicate a better mental health outcome than expected relative to bereavement exposure within this cohort. A negative residual for a bereaved individual, on the other hand, would suggest poorer mental health than expected given their exposure status. With a binary exposure that induces only moderate risk for adverse psychological outcomes, the expected variance explained in our outcome is minimal. Thus, in sensitivity analyses, we added youth’s life events score between 5 and 7 as a continuous covariate in the regression to capture more variance in the stressor exposure. Specifically, the addition of this covariate results in a residual that patterns SDQ response relative to both bereavement and a count of life stressors prior to bereavement.

To define relative cross-domain resilience around age 16, we first obtained normed standardized residuals from a separate regression of each outcome on bereavement for the remaining seven outcomes. We transformed variables with high skew using square root (AUDIT, school attendance, friends) or log (BMI, CRP) transformations. As was done with the SDQ and in order to maintain both strong interpretability (i.e., higher score = stronger resilience) and consistent valence across domains, we negated residuals for AUDIT, blood pressure, BMI, and CRP domains. Finally, we summed an individual’s eight residuals (no weighting scheme used), obtaining a single summary score that captures relative resilience across eight domains. This score assumes that resilience across domains is additive and equally weighted, a convention commonly employed elsewhere in measures such as the Townsend Index [51]. In sensitivity analyses, we explored a seven-domain score that excluded SDQ to identify the extent to which that psychological domain was responsible for driving observed associations with other definitions of resilience.

To compare our continuous approaches to classifying resilience, we estimated correlation coefficients between relative psychological resilience and cross-domain score methods. To compare these two continuous resilience measures with trajectory-based classes, we explored concordance between continuous constructs and class membership using simple percentiles and summary tabulations. Finally, given that these resilience constructs have not yet been applied to a sample of bereaved children, we evaluated the predictive ability of each resilience construct by regressing depressive symptoms at 17.5 years (as measured by MFQ, square root transformed and standardized) on resilience and baseline covariates, one resilience measure at a time, within the bereaved cohort. We expect positive resilience following bereavement to significantly predict lower depressive symptoms later in adolescence, reflecting predictive validity of the construct in question. This validation technique was used previously in the ALSPAC sample with relative resilience applied to adverse childhood experiences exposure [45].

We used multiple imputation with chained equations (m = 10 imputations) to impute all outcome and covariate information among those with bereavement status ascertained. All analyses, unless otherwise specified, drew on these m = 10 imputed datasets, combining results according to Rubin’s Rules [52]. LGMMs were specified using Mplus 8.0 [53] with one randomly selected imputed dataset, as we wanted a consistent imputation model across analyses and Mplus does not support pooling of results from multiply imputed datasets, to our knowledge. All other analyses were run using R 4.1.3 [54].

## Results

Among our analytic sample of N = 3766 youth, 877 (23.3%) were bereaved of a family member between ages 7 and 8.5. Overall, 44.2% of youth were male sex, including 44.7% of non-bereaved youth and 42.6% of bereaved youth (Table 1). Compared to their non-bereaved counterparts, bereaved children exhibited a higher number of stressful life events prior to bereavement (3.2 vs. 2.6), had parents who were less educated (25% vs. 29% of fathers with university degree), and had mothers who were less likely to smoke around birth (9.7% vs. 11.1%); however, these differences were small and largely non-significant.

**Table 1:** Cohort characteristics and missingness

Variable	Original data					Imputed data (M = 10)
	*N*	% Missing	Mean (SD) for continuous variables, % for categorical variables			Mean (SD) for continuous variables, % for categorical variables
			Overall (N = 3769)	Not Bereaved (N = 2891)	Bereaved (N = 878)	
SDQ at 6	3448	8.4%	7.1 (4.6)	7.0 (4.5)	7.3 (4.7)	7.1 (4.6)
SDQ at 9	3668	2.6%	6.4 (4.7)	6.4 (4.6)	6.7 (4.9)	6.4 (4.7)
SDQ at 11	3618	3.9%	6.1 (4.7)	6.1 (4.7)	6.4 (4.8)	6.1 (4.7)
SDQ at 13	3522	6.5%	6.4 (4.7)	6.2 (4.7)	6.7 (4.9)	6.4 (4.8)
SDQ at 16	3276	13.0%	5.8 (4.6)	5.8 (4.5)	6.1 (4.8)	5.9 (4.7)
Frequency (%) attends school at 16	2996	20.4%	94.1 (18.9)	94.2 (18.9)	93.9 (19.3)	93.5 (20.0)
Number of friends	3143	16.5%	7.7 (5.1)	7.7 (5.2)	7.7 (4.9)	7.7 (5.2)
AUDIT score	3175	15.7%	7.0 (4.8)	6.9 (4.8)	7.3 (4.9)	7.0 (4.9)
Blood pressure	3482	7.5%	118.3 (10.6)	118.3 (10.7)	118.3 (10.5)	118.3 (10.7)
Body mass index	3679	2.3%	22.7 (4.1)	22.6 (4.0)	22.9 (4.5)	22.7 (4.1)
C-reactive protein	2456	34.8%	1.5 (3.8)	1.5 (3.8)	1.7 (4.0)	1.6 (4.3)
Hours of sleep, weekday	3094	17.8%	8.3 (0.9)	8.3 (0.9)	8.3 (1.0)	8.2 (0.9)
Life events, 5–7 years	3465	8.0%	2.8 (3.1)	2.6 (3.1)	3.2 (3.3)	2.8 (3.2)
Child sex, male	3766	0.0%	44.2	44.7	42.6	44.2
Mother prenatal financial difficulties	3494	7.2%	2.2 (3.1)	2.2 (3.1)	2.3 (3.1)	2.3 (3.1)
Father educational attainment:	3509	6.8%	–	–	–	–
CSE/none			15.1	14.9	15.5	15.9
Vocational			7.0	7.2	6.3	7.2
O level			21	20.1	23.9	21
A level			29.1	29.2	28.9	28.6
Degree			27.8	28.6	25.3	27.2
Mother educational attainment:	3578	5.0%	–	–	–	–
CSE/none			8.9	8.5	10.2	9.2
Vocational			6.7	6.5	7.5	6.9
O level			33.8	33.1	36.3	33.7
A level			29.2	29.8	26.8	28.8
Degree			21.4	22	19.1	21.3
Maternal smoking at birth	3397	9.8%	10.8	11.1	9.7	11.3
Townsend Index quintile:	2067	45.1%	–	–	–	–
1			31.7	32.0	30.5	32.1
2			15.7	15.5	16.3	15.3
3			19.9	19.5	21.1	20.0
4			23.7	23.8	23.1	23.6
5			9.0	9.1	8.9	8.9

A higher Townsend Index quintile indicates more deprivation

### Trajectory-based psychological resilience

#### Unconditional model

To describe longitudinal change in symptoms of psychopathology among the bereaved cohort, we first estimated a simple growth model without any covariates and compared successive solutions from one to six classes. The three-class model demonstrated superior fit to the two-class solution according to all model fit metrics except for the BLRT (Additional file 2). Additionally, while the four-class model demonstrated marginally lower AIC, BIC, and SSBIC, it exhibited lower entropy and a non-significant BLRT test compared to the three-class solution. Thus, based on fit indices as well as theoretical interpretability, we adopted the three-class solution for subsequent examination in conditional models.

#### Conditional model

**Fig. 2:**
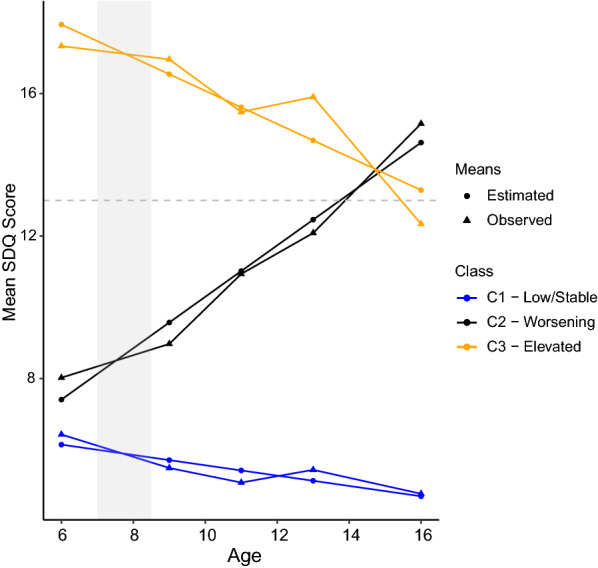
LGMM class mean SDQ trajectories (trajectory-based psychological resilience)

In the next step, we introduced relevant covariates to the unconditional models to adjudicate improvement in model fit. Entropy increased marginally from 0.886 in the unconditional model to 0.889 in the conditional model (Additional file 2). Inclusion of covariates produced class membership probabilities that were nearly identical to the unconditional model. According to this three-class solution, most bereaved youth (N = 741; 84%) demonstrated low and stable SDQ total difficulties scores between ages 6 and 16 (Fig. 2). Only 8% (N = 69) demonstrated worsening SDQ scores over time, and 8% (N = 67) demonstrated improving yet elevated SDQ scores over time.

Finally, we examined covariates that predicted trajectory class assignment. Bereaved children whose mothers had higher prenatal financial difficulties were more likely to belong to the elevated class (vs. the low/stable class; p-value < 0.001; Additional file 3). Males and those with a higher number of stressful life events at 5 were also more likely to belong to the elevated class (p-value = 0.05 for sex; p-value = 0.02 for life events). Bereaved children whose fathers were more educated were more likely to belong to the worsening class (p-value = 0.04), whereas those whose mothers were more educated were less likely to belong to the worsening class (p-value = 0.04). Other covariates included, namely maternal smoking around birth and Townsend Index quintiles, were not significantly associated with class membership among this bereaved cohort. Overall, the trajectory-based approach to defining resilience identified three sub-groups of bereaved youth with distinct psychopathology symptoms over time and with unique sociodemographic characteristics differentiating those three classes.

### Relative psychological resilience

To assess psychological symptoms relative to bereavement exposure, we regressed SDQ total difficulties score at 16 on bereavement exposure between ages 7 and 8.5 and recorded the normed standardized residuals from this regression. On average, bereavement was associated with a 0.08 SD increase in SDQ score at 16 (p-value < 0.05; Additional file 4). Among bereaved individuals, residuals ranged from -3.3 (suggestive of poor resilience relative to this bereaved cohort) to 2.3 (strong resilience). Critically, bereavement exposure explained less than 1% of the variance in SDQ score at 16, resulting in residuals that were nearly perfectly correlated with observed SDQ score (r = − 1.00, p-value < 0.001). Consequently, all interpretations of the relative psychological resilience construct must be made with caution. In sensitivity analyses, regressing SDQ score on bereavement and life events score resulted in similar conclusions but with increased variance explained (Additional file 4). These residuals were also strongly correlated with observed SDQ score (r = − 1.00, p-value < 0.001). Overall, we identified a weak association between bereavement and psychological symptoms, resulting in a resilience score that aligned closely with the psychological symptom score but that did not depend on bereavement status much at all.

### Relative cross-domain resilience

Our relative cross-domain resilience score examined functioning relative to bereavement status across eight unique domains of health. This cross-domain approach revealed wide variability in multi-domain outcomes around age 16 within the bereaved cohort. First, we obtained standardized residuals from independent regressions with the seven additional outcomes, following the same process that we did for psychological functioning. For each domain, bereavement explained minimal variance in the observed outcome, resulting in residuals that were highly correlated with the observed outcome. Overall, relative resilience across these eight domains was at most moderately correlated. Our two markers of resilience in cardiometabolic health (residuals for BMI and blood pressure) were positively corelated (r = 0.32; p-value < 0.05). Body inflammation positively correlated with BMI (r = 0.27; p-value < 0.05), whereas alcohol use and social functioning were negatively correlated (r = − 0.14; p-value < 0.05; Table 2). Weaker correlations existed between other resilience domains, including psychological functioning with school attendance and social functioning, school attendance with alcohol use, and BMI with sleep duration. Standardized residuals across these eight domains were then summed to obtain a cross-domain relative resilience score. Cross-domain resilience was normally distributed, with mean 0 and standard deviation 3.4 among bereaved individuals (Additional file 5). This score covaried most strongly with the BMI domain (r = 0.55; p-value < 0.05) and least strongly with the social functioning domain (r = 0.30; p-value < 0.05). A 7-domain score that excluded psychological functioning was similarly distributed around mean 0, but with smaller standard deviation 3.1 among bereaved individuals (Additional file 6). Correlation between the 7- and 8-domain scores was high at 0.95, as expected. Overall, resilience in one domain of health did not correlate strongly with resilience in other domains of health.

**Table 2:** Cross-Domain Relative Resilience (X Domains)

	Relative Resilience, SDQ										
Relative Resilience, SDQ	1.00	Relative Resilience, School									
Relative Resilience, School	0.11*	1.00	Relative Resilience, Friends								
Relative Resilience, Friends	0.10*	0.03	1.00	Relative Resilience, AUDIT							
Relative Resilience, AUDIT	0.03	0.09*	− 0.14*	1.00	Relative Resilience, BP						
Relative Resilience, BP	0.00	0.00	− 0.07*	0.04*	1.00	Relative Resilience, BMI					
Relative Resilience, BMI	0.05*	0.06*	0.00	0.03	0.32*	1.00	Relative Resilience, CRP				
Relative Resilience, CRP	0.08*	0.01	0.02	0.03	0.05*	0.27*	1.00	Relative Resilience, Sleep			
Relative Resilience, Sleep	0.08*	0.07*	0.05*	0.07*	0.00	0.09*	0.04	1.00	Relative Resilience, SDQ (Adjusted)		
Relative Resilience, SDQ (Adjusted)	1.00*	0.10*	0.10*	0.03	0.00	0.05*	0.08*	0.08*	1.00	Cross-Domain Relative Resilience (8 Domains)	
Cross-Domain Relative Resilience (8 Domains)	0.43*	0.41*	0.30*	0.35*	0.41*	0.55*	0.45*	0.42*	0.43*	1.00	Cross-Domain Relative Resilience (7 Domains)
Cross-Domain Relative Resilience (7 Domains)	0.14*	0.41*	0.30*	0.37*	0.45*	0.59*	0.47*	0.43*	0.14*	0.95*	1.00

For each variable listed above, a higher score reflects higher resilience or a healthier outcome. The adjusted SDQ residual regresses SDQ on bereavement and life events score as a sensitivity analysis. The 7-domain sum score simply excludes SDQ (psychological functioning) as a sensitivity analysis

*p-value < 0.05

### Comparing resilience constructs

We found moderate to low correlation between our two cross-sectional, continuous resilience constructs. Specifically, we found a correlation of 0.43 (p-value < 0.05) between relative psychological resilience at age 16 and the 8-domain relative resilience score around age 16 that includes psychological functioning (Table 2). However, when we excluded psychological functioning from the cross-domain score, the correlation with relative psychological resilience was only 0.14 (p-value < 0.05), suggesting a small but meaningful association between relative psychological resilience and resilience in other domains. The correlation of cross-domain scores with relative psychological resilience adjusted for life events prior to bereavement followed a similar pattern.

Next, we examined the concordance of these constructs in identifying individuals as resilient by comparing the proportion of each latent class in the 50th and 90th percentiles of each continuous resilience construct. Assuming meaningful latent class assignment and continuous resilience constructs, we expect the low/stable psychological symptom class to be over-represented (e.g., > 50% in the 50th percentile) and the other two classes under-represented (e.g., < 10% in the 90th percentile) among the most resilient individuals identified by each continuous resilience construct. In other words, we define concordance here as the extent to which these constructs identify the same set of individuals as resilient or not (i.e., concordance of “resilient” group membership, rather than correlation of outcomes); this assessment will be useful in conjunction with the methodological similarities and differences of each construct. Within the low/stable symptom class, 62% (16%) were in the 50th (90th) percentile of relative psychological resilience scores, and 54% (10%) were in the 50th (90th) percentile of cross-domain scores (Table 3). Within the worsening symptom class, 3% (0%) were in the 50th (90th) percentile of relative psychological resilience scores, and 20% (2%) were in the 50th (90th) percentile of cross-domain scores. Finally, within the elevated symptom class, 11% (0%) were in the 50th (90th) percentile of relative psychological resilience scores, and 22% (3%) were in the 50th (90th) percentile of cross-domain scores. The low/stable symptom class was over-represented and the worsening and elevated symptom classes underrepresented in the top percentiles of both continuous scores, indicating meaningful concordance of trajectory-based resilience with both relative psychological and cross-domain resilience. While both scores exhibited a degree of concordance with class assignment, relative psychological resilience was more concordant with class membership than was relative cross-domain resilience.

**Table 3:** Concordance of continuous resilience constructs and class assignment

	Low/stable class (N = 741, or 84%)	Worsening class (N = 69, or 8%)	Elevated class (N = 67, or 8%)
*Proportion of latent class in 50th percentile of continuous score*			
Relative SDQ resilience	0.62	0.03	0.11
Relative SDQ resilience, adjusted	0.59	0.03	0.08
Cross-domain relative resilience, 8 domains	0.54	0.20	0.22
Cross-domain relative resilience, 7 domains	0.51	0.36	0.33
*Proportion of latent class in 90th percentile of continuous score*			
Relative SDQ resilience	0.16	0.00	0.00
Relative SDQ resilience, adjusted	0.12	0.00	0.00
Cross-domain relative resilience, 8 domains	0.10	0.02	0.03
Cross-domain relative resilience, 7 domains	0.10	0.07	0.06

The adjusted SDQ residual regresses SDQ on bereavement and life events score as a sensitivity analysis. The 7-domain sum score simply excludes SDQ (psychological functioning) from the cross-domain score as a sensitivity analysis. Assuming random assignment of latent class membership and/or poor continuous resilience constructs, we would expect 50% (10%) of each class to be in the 50th (90th) percentile of each continuous resilience construct. Assuming meaningful latent class assignment and continuous resilience constructs, we expect the low/stable class to be over-represented and the other two classes under-represented

Finally, we assessed the predictive validity of each resilience measure by regressing depressive symptoms at 17.5 (as measured by the MFQ) on each resilience construct and other covariates. All resilience measures were significantly predictive of depressive symptoms at age 17.5 (Table 4). Class assignment and relative psychological resilience more strongly predicted depressive symptoms compared to the cross-domain score, though differences in the coefficients of determination were minor. Those in the worsening and elevated classes exhibited more depressive symptoms, on average, compared to those in the low/stable class (worsening class: β = 0.43; p-value < 0.01). Higher relative psychological resilience strongly predicted fewer depressive symptoms as well (β = − 0.19; p-value < 0.001). On the other hand, a one-SD increase in relative cross-domain resilience (8 domains) was only associated with a 0.06 lower square root MFQ score (p-value < 0.001); the association between 7-domain resilience and depressive symptoms at age was very similar.

**Table 4:** Predictive validity of resilience constructs with MFQ at 17.5

Model	Coefficient	Beta	SE	P-value	Model R^2^
MFQ ~ relative psychological resilience + covariates	Relative resilience, SDQ	− 0.19	0.04	< 0.001	0.07
MFQ ~ relative psychological resilience (adjusted) + covariates	Relative resilience, SDQ (adjusted)	− 0.19	0.04	< 0.001	0.07
MFQ ~ cross-domain relative resilience 8 + covariates	Cross-domain relative resilience, 8 domains	− 0.06	0.01	< 0.001	0.06
MFQ ~ cross-domain relative resilience 7 + covariates	Cross-domain relative resilience, 7 domains	− 0.05	0.01	< 0.001	0.05
MFQ ~ class assignment + covariates	LGMM class assignment: worsening class vs. low/stable class	0.43	0.15	< 0.01	0.06
	LGMM class assignment: elevated class vs. low/stable class	0.51	0.15	< 0.001	

Each model regresses MFQ total score (square root transformed due to high skew) on the specified resilience construct and the following covariates: child’s sex (female vs. male), child’s life events score between 5 and 7, maternal financial difficulties at 32 weeks of gestation, paternal and maternal educational attainment, maternal smoking status around birth (yes vs. no), and Townsend deprivation index quintiles

## Discussion

To our knowledge, this is the first study to compare different measures of resilience—trajectory-based psychological resilience, relative psychological resilience, and a relative cross-domain resilience score—among bereaved youth in the same longitudinal dataset. Growth models identified three bereavement response trajectories capturing low/stable (84%), worsening (8%), and elevated (8%) psychological symptom profiles. We found at most moderate correlation (r <  = 0.32) between eight individual resilience domains, low correlation (r = 0.14) between relative psychological resilience and relative cross-domain resilience related to physical and social health (but excluding psychological health), relatively strong concordance between relative psychological resilience and corresponding trajectory classification, and relatively poor concordance between relative cross-domain resilience and trajectory classification. Each resilience measure displayed significant predictive validity with depressive symptoms at age 17.5, though the performance of the relative resilience constructs must be interpreted with caution given the high correlation between residuals and observed outcomes.

Our investigation of trajectory-based psychological resilience in a bereaved youth cohort yielded two key insights that align with extant literature on bereavement and psychopathology. First, trajectory-based results in the present study were consistent with prior work using similar methods among other bereaved adult samples [55–57]. Most bereaved youth in our sample maintained low and stable psychological symptom profiles over time, aligning with prior evidence demonstrating that a significant proportion of bereaved individuals will sustain low symptom trajectories over time [20]. In addition, we note that psychiatric symptoms prior to bereavement–not just at the last assessment point–differentiated psychological functioning trajectories among bereaved individuals rather significantly. This finding is supported by prior studies linking pre-trauma psychopathology to worse post-trauma sequalae and underscores the value of incorporating pre-bereavement risk into assessments of resilience following bereavement [58]. The application of the growth model approach to other domains of health and functioning (e.g., academic performance) may similarly facilitate identification of individuals at-risk for adverse outcomes after bereavement, even before the stressor occurs.

Next, the application of the residual-based approach to defining both psychological and cross-domain resilience highlighted two important limitations of existing approaches to defining resilience after trauma. First, we identify limited use of the residual-based approach to defining resilience in studies where the relationship between exposure and outcome is not meaningfully correlated. In this sample, bereavement was associated with minimal psychological risk, explaining little variance in observed psychological symptoms at age 16 and producing residuals that were nearly perfectly correlated with observed SDQ outcome values. Consequently, relative psychological resilience was just a proxy for the observed SDQ score, and interpretations of concordance and predictive validity shifted away from our resilience target and towards the SDQ scale itself. The low magnitude of association observed between bereavement and psychological symptoms may be explained by our broad definition of bereavement, which included the death of any family member; this limitation is explained in further detail below. Building on prior work that applied this residual-based method, we recommend consistent reporting of the amount of variance explained by the stressor to aid interpretability of findings [21, 29]. We further caution against future application of this resilience construct for a binary trauma exposure that exerts a small effect, as the assumption of linearity between exposure and outcome will likely not be satisfied.

Second, the low correlation observed between relative psychological resilience and the relative cross-domain resilience score illustrates that health in any one resilience domain does not reliably associate with health in a different resilience domain. It is evident that integration of psychological, physical, and social domains of functioning in future studies of resilience is critical to better capturing heterogeneity in post-bereavement well-being. We discourage classification of individuals as resilient based on psychological functioning alone because post-bereavement decrements in health may manifest across social and/or physical domains [33]. To date, recommendations for a more comprehensive assessment of resilience, including examination of a critical set of outcomes or of composite scores, remain empirically understudied [18, 33, 59]. This may be explained, in part, by the methodological complexities of modeling longitudinal, multi-level outcomes [17]. In our analysis, we weighted each resilience domain equally and assumed that domains contributed additively and independently towards the overall cross-domain resilience score. However, a burgeoning literature suggests that adolescents who face adversity yet demonstrate positive psychological adjustment are more likely to exhibit higher physiological stress over time [60, 61]. Accordingly, the influence of these domains may ultimately be modeled differently, with observed correlations and interactions, timing of outcomes assessment, and prior evidence of relative impact all taken into account. From a clinical perspective, this cross-domain heterogeneity suggests that bereaved children may benefit from assessment of social and physical health outcomes even in the absence of mental health symptom endorsement. Youth who appear resilient from a mental health perspective may require services or interventions specific to other key domains of health and functioning.

Numerous study limitations should be considered. First, we were not able to determine the relationship of the study child to the deceased family member, and we expect this relationship to considerably shape subsequent risk of adverse outcomes. For example, we expect the loss of a parent to be more impactful to a child than the loss of a distant relative–and the variance explained in psychiatric symptoms to be larger, as a result. In addition, we could not disentangle time-varying estimates of acute post-bereavement symptoms vs. longer term effects without a more unified timing for our exposure or more outcome measurements. The latter two limitations likely help to explain the modest statistical association observed between bereavement and psychiatric symptoms. Third, the residual approach required outcomes to exhibit positive versus negative functioning valence along just one axis or direction. We selected low (vs. high) BMI and long (vs. short) sleep duration to represent the “healthy” valence or direction. In reality, of course, very low BMI values or very long sleep duration are not necessarily representative of positive functioning. Fourth, prior to our imputation, SDQ scores were prorated, whereas MFQ scores were not. However, given the high internal consistency for both SDQ and MFQ, coupled with our use of imputation for missing values, we do not suspect this to be an important limitation. Finally, we imputed outcomes and covariates using a robust set of auxiliary variables but required bereavement status to be ascertained for analytic sample inclusion, without the use of inverse probability weighting. We cannot rule out selection bias, especially as it relates to bereavement status ascertainment.

In conclusion, we compared three resilience constructs on the same longitudinal data to better understand the distribution of resilience among bereaved youth. Using LGMMs, we found evidence that psychological symptom trajectories among bereaved youth are similar to those identified among bereaved adults and that these trajectories are predictive of depressive symptoms in late adolescence. The residual-based approach, on the other hand, exhibited limited utility and interpretability in the context of bereavement. Despite this limitation, we showed that individual-level resilience varied greatly across domains of health. Our explorations of cross-domain resilience call for better address of the interplay across the psychosocial and physical health domains that shape resilience after trauma. Overall, this study contributes to the growing literature documenting important heterogeneity in the impact of and response to bereavement among youth.

## Supplementary Information

**Additional file 1.** Study Population Flow Chart.

**Additional file 2.** LGMM Model Fit Summary by Class Solution.

**Additional file 3.** LGMM Conditional Model Covariates.* p-value < 0.05. All covariates were entered numerically. A higher Townsend Index quintile indicates more deprivation, whereas a higher education score reflects more education.

**Additional file 4.** Relative Resilience Model Results of SDQ at 16 and Bereavement. Outcome was standardized to have mean 0 and SD 1 after square root transformation; estimates are reported in these units. Standardized residuals (but not the model coefficients) were negated so that a positive residual suggests a positive health outcome.

**Additional file 5.** Distribution of Cross-Domain Scores (8 Domains) Among Bereaved YPs. Distribution of scores is based on randomly selected imputation #7. A higher score reflects higher cross-domain resilience.

**Additional file 6.** Distribution of Cross-Domain Scores (7 Domains) Among Bereaved YPs (Sensitivity Analysis Excluding SDQ from Sum Score). Distribution of scores is based on randomly selected imputation #7. A higher score reflects higher cross-domain resilience.

## Data Availability

Data are from ALSPAC and are not publicly available but may be made available upon request to the ALSPAC Study Team. Further information, including the procedures to obtain and access data, is described at http://www.bristol.ac.uk/alspac/researchers/access/.

## Abbreviations

AIC: Akaike information criterion
ALSPAC: Avon Longitudinal Study of Parents and Children
AUDIT: Alcohol Use Disorders Identification Test
BIC: Bayesian information criterion
BLRT: Bootstrap Likelihood Ratio Test
BMI: Body mass index
CRP: C-reactive protein
CSE: Certificate of Secondary Education
LGMM: Latent growth mixture model
LMR-LRT: Lo-Mendell-Rubin Likelihood Ratio Test
MFQ: Moods and Feelings Questionnaire
SD: Standard deviation
SDQ: Strengths and Difficulties Questionnaire
SSBIC: Sample size-adjusted Bayesian information criterion

## References

[1] Benjet C, Bromet E, Karam EG, Kessler RC, McLaughlin KA, Ruscio AM (2016). The epidemiology of traumatic event exposure worldwide: results from the World Mental Health Survey Consortium. Psychol Med.

[2] Keyes KM, Pratt C, Galea S, McLaughlin KA, Koenen KC, Shear MK (2014). The burden of loss: unexpected death of a loved one and psychiatric disorders across the life course in a national study. Am J Psychiatry.

[3] Oh DL, Jerman P, Silvério Marques S, Koita K, Purewal Boparai SK, Burke Harris N (2018). Systematic review of pediatric health outcomes associated with childhood adversity. BMC Pediatr.

[4] Petruccelli K, Davis J, Berman T (2019). Adverse childhood experiences and associated health outcomes: a systematic review and meta-analysis. Child Abuse Negl.

[5] Applebaum DR, Burns GL (1991). Unexpected childhood death: posttraumatic stress disorder in surviving siblings and parents. J Clin Child Psychol.

[6] Carr D, Boerner K, Moorman S (2020). Bereavement in the time of coronavirus: unprecedented challenges demand novel interventions. J Aging Soc Policy.

[7] Barenbaum E, Smith T (2016). Social support as a protective factor for children impacted by HIV/AIDS across varying living environments in southern Africa. AIDS Care.

[8] Kaplow JB, Saunders J, Angold A, Costello EJ (2010). Psychiatric symptoms in bereaved versus nonbereaved youth and young adults: a longitudinal epidemiological study. J Am Acad Child Adolesc Psychiatry.

[9] Harrison L, Harrington R (2001). Adolescents’ bereavement experiences. Prevalence, association with depressive symptoms, and use of services. J Adolesc.

[10] Guldin M-B, Li J, Pedersen HS, Obel C, Agerbo E, Gissler M (2015). Incidence of suicide among persons who had a parent who died during their childhood: a population-based cohort study. JAMA Psychiat.

[11] Bergman A-S, Axberg U, Hanson E (2017). When a parent dies—a systematic review of the effects of support programs for parentally bereaved children and their caregivers. BMC Palliat Care.

[12] Pham S, Porta G, Biernesser C, Walker Payne M, Iyengar S, Melhem N (2018). The burden of bereavement: early-onset depression and impairment in youths bereaved by sudden parental death in a 7-year prospective study. Am J Psychiatry.

[13] Alciati A, Gesuele F, Casazza G, Foschi D (2013). The relationship between childhood parental loss and metabolic syndrome in obese subjects. Stress Health.

[14] Dietz LJ, Stoyak S, Melhem N, Porta G, Matthews KA, Walker Payne M (2013). Cortisol response to social stress in parentally bereaved youth. Biol Psychiatry.

[15] Shear MK (2012). Getting straight about grief. Depress Anxiety.

[16] Bonanno GA (2004). Loss, trauma, and human resilience: have we underestimated the human capacity to thrive after extremely aversive events?. Am Psychol.

[17] Cicchetti D (2010). Resilience under conditions of extreme stress: a multilevel perspective. World Psychiatry.

[18] Denckla CA, Cicchetti D, Kubzansky LD, Seedat S, Teicher MH, Williams DR (2020). Psychological resilience: an update on definitions, a critical appraisal, and research recommendations. Eur J Psychotraumatol.

[19] Bonanno GA, Wortman CB, Lehman DR, Tweed RG, Haring M, Sonnega J (2002). Resilience to loss and chronic grief: a prospective study from preloss to 18-months postloss. J Pers Soc Psychol.

[20] Djelantik AAAMJ, Robinaugh DJ, Boelen PA (2022). The course of symptoms in the first 27 months following bereavement: a latent trajectory analysis of prolonged grief, posttraumatic stress, and depression. Psychiatry Res.

[21] Nishimi K, Choi KW, Cerutti J, Powers A, Bradley B, Dunn EC (2021). Measures of adult psychological resilience following early-life adversity: how congruent are different measures?. Psychol Med.

[22] Bonanno GA, Neria Y, Mancini A, Coifman KG, Litz B, Insel B (2007). Is there more to complicated grief than depression and posttraumatic stress disorder? A test of incremental validity. J Abnorm Psychol.

[23] Shear MK, Simon N, Wall M, Zisook S, Neimeyer R, Duan N (2011). Complicated grief and related bereavement issues for DSM-5. Depress Anxiety.

[24] Luecken LJ, Roubinov DS (2012). Pathways to lifespan health following childhood parental death. Soc Personal Psychol Compass.

[25] Richman LS, Kubzansky L, Maselko J, Kawachi I, Choo P, Bauer M (2005). Positive emotion and health: going beyond the negative. Health Psychol.

[26] VanderWeele TJ, Chen Y, Long K, Kim ES, Trudel-Fitzgerald C, Kubzansky LD (2020). Positive epidemiology?. Epidemiology.

[27] Bonanno GA, Westphal M, Mancini AD (2011). Resilience to loss and potential trauma. Annu Rev Clin Psychol.

[28] Kim-Cohen J, Moffitt TE, Caspi A, Taylor A (2004). Genetic and environmental processes in young children’s resilience and vulnerability to socioeconomic deprivation. Child Dev.

[29] Amstadter AB, Myers JM, Kendler KS (2014). Psychiatric resilience: longitudinal twin study. Br J Psychiatry.

[30] Choi KW, Stein MB, Dunn EC, Koenen KC, Smoller JW (2019). Genomics and psychological resilience: a research agenda. Mol Psychiatry.

[31] Ryan M, Ryznar R (2022). The molecular basis of resilience: a narrative review. Front Psychiatry.

[32] Luthar SS, Doernberger CH, Zigler E (1993). Resilience is not a unidimensional construct: insights from a prospective study of inner-city adolescents. Dev Psychopathol.

[33] Luthar SS, Cicchetti D, Becker B (2000). The construct of resilience: a critical evaluation and guidelines for future work. Child Dev.

[34] Boyd A, Golding J, Macleod J, Lawlor DA, Fraser A, Henderson J (2013). Cohort Profile: the ’children of the 90s’—the index offspring of the Avon Longitudinal Study of Parents and Children. Int J Epidemiol.

[35] Fraser A, Macdonald-Wallis C, Tilling K, Boyd A, Golding J, Davey Smith G (2013). Cohort profile: the avon longitudinal study of parents and children: ALSPAC mothers cohort. Int J Epidemiol.

[36] Golding J, Pembrey M, Jones R, ALSPAC Study Team (2001). ALSPAC–the Avon Longitudinal Study of Parents and Children. I. Study methodology. Paediatr Perinat Epidemiol.

[37] Cornish RP, Macleod J, Boyd A, Tilling K (2021). Factors associated with participation over time in the Avon Longitudinal Study of Parents and Children: a study using linked education and primary care data. Int J Epidemiol.

[38] Dohrenwend BP (2006). Inventorying stressful life events as risk factors for psychopathology: toward resolution of the problem of intracategory variability. Psychol Bull.

[39] Goodman R (1997). The strengths and difficulties questionnaire: a research note. J Child Psychol Psychiatry.

[40] Angold A, Costello EJ, Messer SC, Pickles A, Winder F, Silver D (1995). Development of a short questionnaire for use in epidemiological studies of depression in children and adolescents. Int J High Risk Behav Addict.

[41] Babor TF, Higgins-Biddle JC, Saunders JB, Monteiro MG. The alcohol use disorders identification test. http://www.psiholocator.com/images/who_msd_msb_016a.pdf. Accessed 22 Jul 2022.

[42] Smerillo NE, Reynolds AJ, Temple JA, Ou S-R (2018). Chronic absence, eighth-grade achievement, and high school attainment in the Chicago Longitudinal Study. J Sch Psychol.

[43] Ni Mhurchu C, Turley M, Gorton D, Jiang Y, Michie J, Maddison R (2010). Effects of a free school breakfast programme on school attendance, achievement, psychosocial function, and nutrition: a stepped wedge cluster randomised trial. BMC Public Health.

[44] Schultebraucks K, Choi KW, Galatzer-Levy IR, Bonanno GA (2021). Discriminating heterogeneous trajectories of resilience and depression after major life stressors using polygenic scores. JAMA Psychiat.

[45] Cahill S, Hager R, Chandola T (2022). The validity of the residuals approach to measuring resilience to adverse childhood experiences. Child Adolesc Psychiatry Ment Health.

[46] Hirshkowitz M, Whiton K, Albert SM, Alessi C, Bruni O, DonCarlos L (2015). National Sleep Foundation’s sleep time duration recommendations: methodology and results summary. Sleep Health.

[47] Weinberg RJ, Dietz LJ, Stoyak S, Melhem NM, Porta G, Payne MW (2013). A prospective study of parentally bereaved youth, caregiver depression, and body mass index. J Clin Psychiatry.

[48] Jung T, Wickrama KAS (2008). An introduction to latent class growth analysis and growth mixture modeling. Soc Personal Psychol Compass.

[49] Lo Y, Mendell NR, Rubin DB (2001). Testing the number of components in a normal mixture. Biometrika.

[50] Herle M, Stavola BD, Hübel C, Abdulkadir M, Ferreira DS, Loos RJF (2020). A longitudinal study of eating behaviours in childhood and later eating disorder behaviours and diagnoses. Br J Psychiatry.

[51] Sanah Yousaf AB. UK Townsend Deprivation Scores from 2011 census data. 2017. http://s3-eu-west-1.amazonaws.com/statistics.digitalresources.jisc.ac.uk/dkan/files/Townsend_Deprivation_Scores/UK%20Townsend%20Deprivation%20Scores%20from%202011%20census%20data.pdf.

[52] Rubin DB (1987). Multiple imputation for nonresponse in surveys.

[53] Muthen LK, Muthen B (2017). Mplus Version 8 user’s guide.

[54] Computing. R: a language and environment for statistical computing. Vienna: R Core Team. https://www.yumpu.com/en/document/view/6853895/r-a-language-and-environment-for-statistical-computing.

[55] Lundorff M, Bonanno GA, Johannsen M, O’Connor M (2020). Are there gender differences in prolonged grief trajectories? A registry-sampled cohort study. J Psychiatr Res.

[56] Mancini AD, Sinan B, Bonanno GA (2015). Predictors of prolonged grief, resilience, and recovery among bereaved spouses. J Clin Psychol.

[57] Maccallum F, Galatzer-Levy IR, Bonanno GA (2015). Trajectories of depression following spousal and child bereavement: a comparison of the heterogeneity in outcomes. J Psychiatr Res.

[58] Gradus JL, Rosellini AJ, Szentkúti P, Horváth-Puhó E, Smith ML, Galatzer-Levy I (2022). Pre-trauma predictors of severe psychiatric comorbidity 5 years following traumatic experiences. Int J Epidemiol.

[59] Jaffee SR, Caspi A, Moffitt TE, Polo-Tomás M, Taylor A (2007). Individual, family, and neighborhood factors distinguish resilient from non-resilient maltreated children: a cumulative stressors model. Child Abuse Negl.

[60] De France K, Evans GW, Brody GH, Doan SN (2022). Cost of resilience: Childhood poverty, mental health, and chronic physiological stress. Psychoneuroendocrinology.

[61] Miller GE, Yu T, Chen E, Brody GH (2015). Self-control forecasts better psychosocial outcomes but faster epigenetic aging in low-SES youth. Proc Natl Acad Sci U S A.

