# Correction: Resilient phenotypes among bereaved youth: a comparison of trajectory, relative, and cross-domain approaches

**DOI:** 10.1186/s13034-023-00582-2

**Published:** 2023-03-14

**Authors:** Ana Lucia Espinosa Dice, Xian Ye, Stephanie Gyuri Kim, Katie A. McLaughlin, Ananda B. Amstadter, Henning Tiemeier, Christy A. Denckla

**Affiliations:** 1 Department of Epidemiology, Harvard T.H. Chan School of Public Health, Boston, MA USA; 2 Department of Biostatistics, Harvard T.H. Chan School of Public Health, Boston, MA USA; 3 Human Development and Family Studies, University of Illinois Urbana-Champaign, Champaign, IL USA; 4 Department of Psychology, Harvard University, Cambridge, MA USA; 5 Virginia Institute for Psychiatric and Behavioral Genetics, Virginia Commonwealth University, Richmond, VA USA; 6 Department of Social and Behavioral Sciences, Harvard T.H. Chan School of Public Health, Boston, MA USA

**Correction: Child and Adolescent Psychiatry and Mental Health (2023) 17:23** **https://doi.org/10.1186/s13034-023-00568-0**

Following publication of the original article, the author noticed the induced errors occurred in the published version. The typesetter has inadvertently removed the captions of Figure 2 and Table 2 during correction stage. Figure 2 and Table 2 along with the captions have been presented with this correction.

**Fig. 2:**
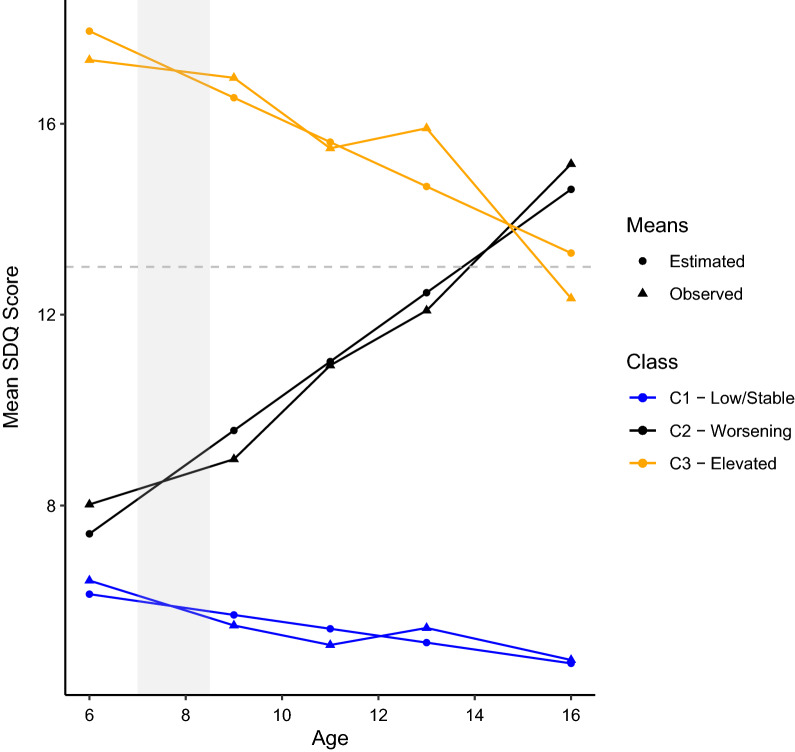
LGMM class mean SDQ trajectories (trajectory-based psychological resilience). A higher SDQ score reflects worse emotional and behavioral difficulties. Grey line at 13 separates normal scores (≤13) from borderline/abnormal scores (>13) according to the parent completed SDQ. Grey shaded region reflects timeline of bereavement exposure

**Table 2:** Correlation Coefficients Between Continuous Resilience Constructs (Relative Psychological and Cross-Domain Resilience)

	**Relative Resilience, SDQ**										
**Relative Resilience, SDQ**	1	**Relative Resilience, School**									
**Relative Resilience, School**	0.11*	1	**Relative Resilience, Friends**								
**Relative Resilience, Friends**	0.10*	0.03	1	**Relative Resilience, AUDIT**							
**Relative Resilience, AUDIT**	0.03	0.09*	-0.14*	1	**Relative Resilience, BP**						
**Relative Resilience, BP**	0	0	-0.07*	0.04*	1	**Relative Resilience, BMI**					
**Relative Resilience, BMI**	0.05*	0.06*	0	0.03	0.32*	1	**Relative Resilience, CRP**				
**Relative Resilience, CRP**	0.08*	0.01	0.02	0.03	0.05*	0.27*	1	**Relative Resilience, Sleep**			
**Relative Resilience, Sleep**	0.08*	0.07*	0.05*	0.07*	0	0.09*	0.04	1	**Relative Resilience, SDQ (Adjusted)**		
**Relative Resilience, SDQ (Adjusted)**	1.00*	0.10*	0.10*	0.03	0	0.05*	0.08*	0.08*	1	**Cross-Domain Relative Resilience (8 Domains)**	
**Cross-Domain Relative Resilience (8 Domains)**	0.43*	0.41*	0.30*	0.35*	0.41*	0.55*	0.45*	0.42*	0.43*	1	**Cross-Domain Relative Resilience (7 Domains)**
**Cross-Domain Relative Resilience (7 Domains)**	0.14*	0.41*	0.30*	0.37*	0.45*	0.59*	0.47*	0.43*	0.14*	0.95*	1

For each variable listed above, a higher score reflects higher resilience or a healthier outcome. The adjusted SDQ residual regresses SDQ on bereavement and life events score as a sensitivity analysis. The 7-domain sum score simply excludes SDQ (psychological functioning) as a sensitivity analysis

* p-value < 0.05.

The original article has been corrected.
