# Supplementary material for: Resilient phenotypes among bereaved youth: a comparison of trajectory, relative, and cross-domain approaches
Source: Child Adolesc Psychiatry Ment Health. 2023 Feb 8;17:23. doi: 10.1186/s13034-023-00568-0 (PMC9909953; doi:10.1186/s13034-023-00568-0)
Supplement: Supplementary file 1 — Additional file 1. Study Population Flow Chart. [file 13034_2023_568_MOESM1_ESM.pdf]

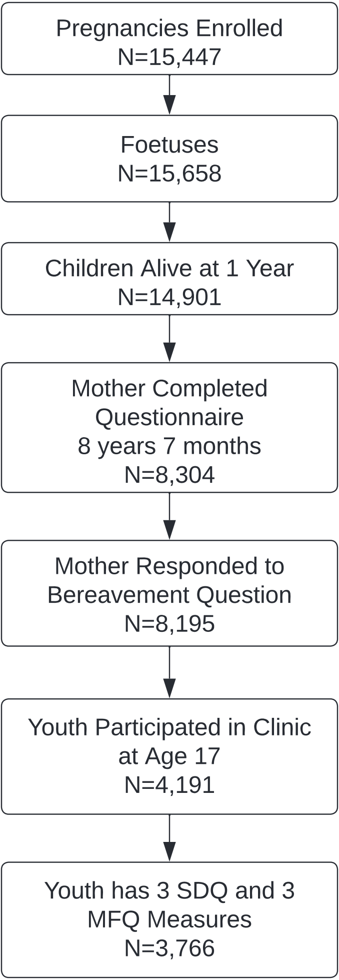
Pregnancies Enrolled
N=15,447
Foetuses
N=15,658
Children Alive at 1 Year
N=14,901
Mother Completed Questionnaire
8 years 7 months
N=8,304
Mother Responded to Bereavement Question
N=8,195
Youth Participated in Clinic at Age 17
N=4,191
Youth has 3 SDQ and 3 MFQ Measures
N=3,766
