# Supplementary material for: Resilient phenotypes among bereaved youth: a comparison of trajectory, relative, and cross-domain approaches
Source: Child Adolesc Psychiatry Ment Health. 2023 Feb 8;17:23. doi: 10.1186/s13034-023-00568-0 (PMC9909953; doi:10.1186/s13034-023-00568-0)
Supplement: Supplementary file 2 — Additional file 2. LGMM Model Fit Summary by Class Solution. [file 13034_2023_568_MOESM2_ESM.docx]

**LGMM Model Fit Summary by Class Solution**

|  | **Fit Measure** | **Number of Classes** | | | | | |
| --- | --- | --- | --- | --- | --- | --- | --- |
| **Model** |  | **One** | **Two** | **Three** | **Four** | **Five** | **Six** |
| **Unconditional** | **AIC** | 23768.585 | 23604.563 | 23466.832 | 23402.000 | 23377.630 | 23360.892 |
|  | **BIC** | 23806.797 | 23657.105 | 23533.703 | 23483.201 | 23473.160 | 23470.752 |
|  | **SSBIC** | 23781.391 | 23622.171 | 23489.242 | 23429.213 | 23409.645 | 23397.709 |
|  | **Entropy** | - | 0.884 | 0.886 | 0.822 | 0.828 | 0.827 |
|  | **LMR-LRT p-value** | - | 0.031 | 0.001 | 0.009 | 0.028 | 0.101 |
|  | **BLRT p-value** | - | 0.000 | 0.667 | 1.000 | 1.000 | 1.000 |
| **Conditional** | **AIC** | 23710.377 | 23582.845 | 23444.241 | 23380.132 | 23359.671 | 23353.452 |
|  | **BIC** | 23815.460 | 23668.822 | 23577.983 | 23561.639 | 23588.943 | 23630.489 |
|  | **SSBIC** | 23745.593 | 23611.658 | 23489.061 | 23440.959 | 23436.506 | 23446.294 |
|  | **Entropy** | - | 0.880 | 0.889 | 0.815 | 0.827 | 0.857 |
|  | **LMR-LRT p-value** | - | 0.051 | 0.000 | 0.047 | 0.069 | 0.240 |
|  | **BLRT p-value** | - | 0.000 | 0.000 | 0.000 | 0.208 | 0.000 |
