# Supplementary material for: Resilient phenotypes among bereaved youth: a comparison of trajectory, relative, and cross-domain approaches
Source: Child Adolesc Psychiatry Ment Health. 2023 Feb 8;17:23. doi: 10.1186/s13034-023-00568-0 (PMC9909953; doi:10.1186/s13034-023-00568-0)
Supplement: Supplementary file 3 — Additional file 3. LGMM Conditional Model Covariates.* p-value < 0.05. All covariates were entered numerically. A higher Townsend Index quintile indicates more deprivation, whereas a higher education score reflects more education. [file 13034_2023_568_MOESM3_ESM.docx]

**LGMM Conditional Model Covariates**

| Covariates | Worsening Class (vs. Low/Stable Class) | | Elevated Class (vs. Low/Stable Class) | |
| --- | --- | --- | --- | --- |
|  | OR | SE | OR | SE |
| Life events score at 5 | 1.05 | 0.05 | 1.10* | 0.05 |
| Child sex (female vs. male) | 1.52 | 0.48 | 0.54* | 0.17 |
| Maternal prenatal financial difficulties | 1.07 | 0.05 | 1.16* | 0.05 |
| Father's educational attainment | 1.38* | 0.21 | 0.78 | 0.11 |
| Mother's educational attainment | 0.73* | 0.11 | 0.93 | 0.15 |
| Maternal smoking around birth (yes vs. no) | 0.87 | 0.43 | 1.46 | 0.63 |
| Townsend Index quintiles | 1.12 | 0.11 | 0.97 | 0.11 |

* p-value < 0.05. All covariates were entered numerically. A higher Townsend Index quintile indicates more deprivation, whereas a higher education score reflects more education.
