# Supplementary material for: Resilient phenotypes among bereaved youth: a comparison of trajectory, relative, and cross-domain approaches
Source: Child Adolesc Psychiatry Ment Health. 2023 Feb 8;17:23. doi: 10.1186/s13034-023-00568-0 (PMC9909953; doi:10.1186/s13034-023-00568-0)
Supplement: Supplementary file 4 — Additional file 4. Relative Resilience Model Results of SDQ at 16 and Bereavement. Outcome was standardized to have mean 0 and SD 1 after square root transformation; estimates are reported in these units. Standardized residuals (but not the model coefficients) were negated so that a positive residual suggests a positive health outcome. [file 13034_2023_568_MOESM4_ESM.docx]

**Relative Resilience Model Results of SDQ at 16 and Bereavement**

| **Model** | **Beta** | **95% CI** | **P-value** | **R-squared** | **Minimum residual among bereaved individuals** | **Maximum residual among bereaved individuals** |
| --- | --- | --- | --- | --- | --- | --- |
| **Crude (SDQ ~ Bereavement)** | 0.077 | 0.001, 0.154 | 0.047 | 0.001 | -3.326 | 2.307 |
| **Adjusted (SDQ ~ Bereavement + Life Events)** | 0.061 | -0.015, 0.138 | 0.116 | 0.009 | -3.388 | 2.442 |

Outcome was standardized to have mean 0 and SD 1 after square root transformation; estimates are reported in these units. Standardized residuals (but not the model coefficients) were negated so that a positive residual suggests a positive health outcome.
