# Supplementary figures and images for: Resilient phenotypes among bereaved youth: a comparison of trajectory, relative, and cross-domain approaches
Source: Child Adolesc Psychiatry Ment Health. 2023 Feb 8;17:23. doi: 10.1186/s13034-023-00568-0 (PMC9909953; doi:10.1186/s13034-023-00568-0)

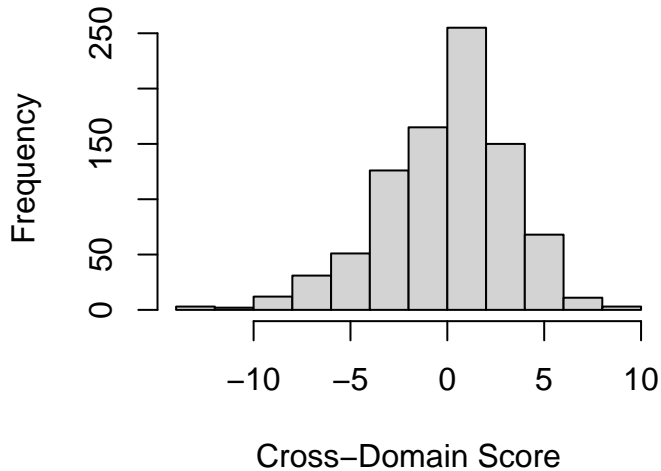

Supplement: Supplementary file 5 — Additional file 5. Distribution of Cross-Domain Scores (8 Domains) Among Bereaved YPs. Distribution of scores is based on randomly selected imputation #7. A higher score reflects higher cross-domain resilience. [file 13034_2023_568_MOESM5_ESM.pdf]

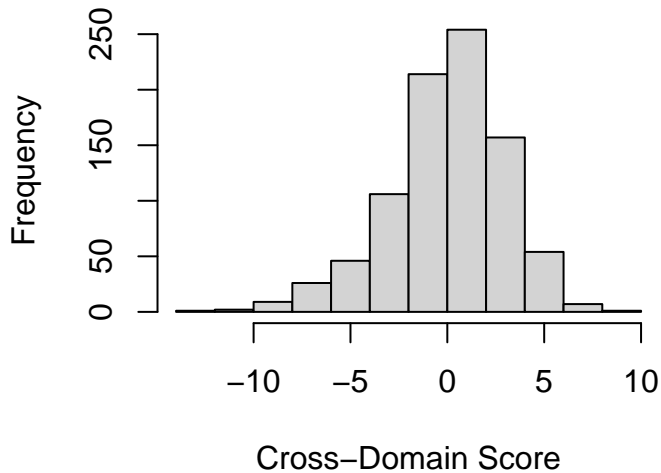

Supplement: Supplementary file 6 — Additional file 6. Distribution of Cross-Domain Scores (7 Domains) Among Bereaved YPs (Sensitivity Analysis Excluding SDQ from Sum Score). Distribution of scores is based on randomly selected imputation #7. A higher score reflects higher cross-domain resilience. [file 13034_2023_568_MOESM6_ESM.pdf]
